# On Elevating the Dental Profession

**Published:** 1870-08

**Authors:** W. E. Driscoll


### ON ELEVATING THE DENTAL PROFESSION.

BY W. E. DRISCOLL.

Read before the Indiana State Dental Association.

The object of this Association is "to contribute to the elevation of the Dental Profession in Indiana," "to promote the usefulness, honor, and interests of its members," "and to advance the standard of Dental education." My object is to contribute what I can in furtherance of the declared purpose of the Association.

There seems to be some question as to what proportion of those trying to practice, should be reckoned in the profession; which question, I think, depends for solution, mainly on the following conditions:

1st. If there is to be no more rapid advancement or elevation of those most in need of such change, than at present seems the rule, many must be counted out, who, under a wiser order of things, might soon be a credit to the profession.

By the present order, I mean that in which a young man is enticed into a Dental office, and persuaded that he can become a full-fledged Dentist in three or six months; after which he may blunder along for an indefinite time, without the slightest suspicion that he lacks anything at all necessary to the successful practice of Dentistry. But, if accidentally, he encounters any of the current Dental literature, he finds himself or his class of Dentists uniformly denominated quacks, charlatans, or brazen impostors, and that too, when possibly the profession does not contain a more conscientious or honest man. His reading may bring him upon an argument that great distinction lay in the ability to swage a plate for artificial teeth, and solder the teeth on it; yet, when he recollects the many inferior operations he has examined, performed by men who apply these harsh terms, he will, if not possessed of greater discretion than his accusers, honestly believe them to be the worst kind of humbugs and pretenders, and will not realize the necessity of any imitation of those against whom he is thus unfortunately prejudiced, and in avoiding them may disregard the opportunity of imitating their genuine acquirements.

By a wiser order of things, I mean that in which the better qualified members extend all the encouragement they can to those of inferior acquirements, on condition that they should unite in support of a law of the State so graduated in its requirements, that all might in a reasonable time, attain to the standard it establishes, and thus do away with all difficulty about who should be recognized as *in* the profession.

As long as the present degree of antagonism and misunderstanding continues between the educated and the uneducated practitioners of Dentistry, all attempts to elevate our specialty to an equality with other professions in the estimation of the world at large, will fail. If there was any way to stop those of inferior qualifications now practicing, that of course would be an end to the matter, as it would leave only those of whose title to professional recognition there would be no question. This it is thought can be substantially effected by the passage and enforcement of a law by the State. Any attempt so far made to secure the passage of such a law in this State has failed, and the indications are that such will continue to be our experience, so long as the measure receives the support of only a mere fraction of our practitioners. And for this reason our legislators will doubt whether it is sound policy to pass a law that so few in the business seem to desire.

They will judge it an unnecessary if not an unjust discrimination, and what will be still more potent with them, they will conclude that the people will be opposed to a law tending to arrest competition in rates for Dental services. I know a very large portion of the people are opposed to such a law as was defeated at the last session of our legislature, intended to regulate the practice of medicine. You can not find the man who does not consider himself capable of selecting his own physician, and the fact of a few practicing without diplomas, is considered by many as an excellent thing inasmuch as it opens the door to greater rivalry, and consequent lower fees. All these objections will be urged against a law to regulate the practice of Dentistry in popular estimation with double force.

In view then of these facts, I ask, is it reasonable to expect or hope for the enactment of a law through the efforts of a small minority of those in practice, and against the probable opposition of a great portion of the rest? But if passed, who would expect to see it successfully enforced, when there could be found only a few isolated graduates and old practitioners who would do anything to make its power felt? Then possibly only enough to cause its repeal by the next legislature. I am informed that the law once enacted in this State, to regulate the practice of medicine, had long been a dead letter when repealed. I am opposed to wasting our time and opportunities, in abortive efforts for a law that will not be enforced, when a better one that will secure its own enforcement can be passed with perfect certainty at the next session of the legislature. Such a law need not be very different from the one proposed, or from those now under experiment in Ohio and New York.

I would at present suggest only this change, that if a very advanced standard should be adopted, by which all must be tried, let those now in practice who desire it, have, say, six years to prepare for the final examination; but, in the meantime, be required to pass examinations once in two years, upon a proportionate part of the ultimate requirement of the law. I would make these provisions so that every one worthy of any consideration, would rather prepare for the different examinations, than to try to break down or evade the law.

As I have before intimated, I consider the *manner* in which this law is brought into existence, is a matter of as much importance as the law itself; since its enactment, retention on the statute books, and its enforcement all depends upon its almost unanimous support of those now practicing Dentistry. And I believe, we can secure for it the support of almost every located Dentist in the State. And this is one way it might be done: Draft such a bill as we desire for a law, also an address to the Dentists of the State, presenting the matter in its proper light, and urging them to use their influence with the Senator and Representative of their respective districts or counties to vote for the bill when presented to the legislature. Place a copy in the hand of every Dentist in the State. If he approves the plan, let him return his endorsement to the committee having charge of the matter, for use at the next session of the legislature. We thus secure the commitment of many to the law, by presenting to them first its advantages; whereas if they were ignored until the law is enforced, would find themselves outlawed, which would so arouse their indignation and prejudice, as to induce them to do all they could to evade it until they could secure its repeal, and that too, with the chances decidedly in their favor.

To some these may seem as useless precautions, but I assure you, there is a strong, decided prejudice among the people, and among Dentists too, against a law like this. I admit it is mainly because of a false view of the matter. The people think if there is no cheap competition, we will be extortionate in our fees. There are those practicing who would do much against it through fear that they would fail to secure their license, and thus be driven from the business, and could make much trouble by leading the people to believe that they were fighting their battle against extortion and humbugs; while really they would be doing exactly the reverse.

So the more we reflect, the clearer is the conviction that we must have unity of action.

It is but fair to infer from the language of some, that they would oppose even the temporary and partial recognition of any one without a diploma. If such council is to prevail, matters will continue just about as they are, and the elevation of the profession will not be visible in this country or generation.

The people will recognize them if we do not, and we as well as the people will suffer more from such a policy, than they from being counted out of the profession, or unfit to unite with us for general elevation.

Almost all contribute something to foreign missions—have we nothing for our own country, or our own profession? Can we not overcome our dislike for some because they do cheap work? It is not always their fault. There is no more propriety in making a person a set of teeth on gold or platinum, when he will only pay for rubber or adamantine, than in giving him a suit of broadcloth, when he calls and pays only for jeans or shoddy.

There are poor people to be supplied with teeth as well as rich ones; and in some country practices, there are none but the poor. Are we to declare against supplying these, or for giving them the best without considering the price they can pay? Such procedure would have about the same influence as the Pope's bull against the comet.

But let us suppose that better counsel will prevail; that all respectable men trying to practice our specialty, will be invited and prevailed upon to unite with us upon such a law, then who can doubt the result?

If it provides for District Societies, as the one in New York, instead of one or two weak and languid Societies in the State, regarded with indifference by most, and with contempt by many, we would have near a score if needed for convenience, and their membership would comprise every Dentist in the State.

The excellent influence such Societies have upon the social relation of members, will not be then confined to so few, and will do more to allay misunderstanding, prejudice, unworthy competition, and ruinous undercharging, than any other means at our command.

Few realize how narrow and selfish were their aims until brought in contact with men imbued with the noble fraternal spirit that prevails at our Society meetings. And few, I hope, ever attend without returning home determined upon a higher stand as men and Dentists.

Very much is gained in the way of mutual respect, by acquaintance with each other. When a man has extended the courtesies of a gentleman, we feel bound to regard him in a favorable light, and will be slower to entertain suspicion of him or to give expression to one.

Then after all have attained the standard the law establishes, these Societies which it will assure to us, will be the means as well as the incentive to our continued steady advancement, far beyond any criterion that would be expedient to anybody in a law.

The hope of our Dental periodicals is in the success of the Societies. The progress of Dental science is so steady, that one who does not read nearly all Dental literature, scarcely dares to engage in the discussion of any subject pertaining to Dentistry.

And lastly, the law and its Societies will be to the Dental colleges, as the spring sunshine and shower to the vegetable world. They will spring at once into a newness of life and vigor heretofore unknown.

In conclusion, every interest of our profession, every hope for a higher and better order of things than at present prevails, is centered in this law, and its adoption in such a manner, as to command the support of the profession.

